# Image metric-based multi-observation single-step deep deterministic policy gradient for sensorless adaptive optics

**DOI:** 10.1364/BOE.528579

**Published:** 2024-07-23

**Authors:** Guozheng Xu, Thomas J. Smart, Eduard Durech, Marinko V. Sarunic

**Affiliations:** 1 Department of Medical Physics and Biomedical Engineering, University College London, London WC1E 6BT, United Kingdom; 2 Institute of Ophthalmology, University College London, London WC1E 6BT, United Kingdom; 3 School of Engineering Science, Simon Fraser University, Burnaby BC V5A 1S6, Canada

## Abstract

Sensorless adaptive optics (SAO) has been widely used across diverse fields such as astronomy, microscopy, and ophthalmology. Recent advances have proved the feasibility of using the deep deterministic policy gradient (DDPG) for image metric-based SAO, achieving fast correction speeds compared to the coordinate search Zernike mode hill climbing (ZMHC) method. In this work, we present a multi-observation single-step DDPG (MOSS-DDPG) optimization framework for SAO on a confocal scanning laser ophthalmoscope (SLO) system with particular consideration for applications in preclinical retinal imaging. MOSS-DDPG optimizes *N* target Zernike coefficients in a single-step manner based on 2*N* + 1 observations of the image sharpness metric values. Through *in silico* simulations, MOSS-DDPG has demonstrated the capability to quickly achieve diffraction-limited resolution performance with long short-term memory (LSTM) network implementation. *In situ* tests suggest that knowledge learned through simulation adapts swiftly to imperfections in the real system by transfer learning, exhibiting comparable *in situ* performance to the ZMHC method with a greater than tenfold reduction in the required number of iterations.

## 1. Introduction

Optical aberrations detrimentally affect image quality by reducing contrast and resolution. Although careful design and alignment of an optical system can minimize system aberrations, for applications such as ocular imaging and deep tissue microscopy, the sample itself is a source of aberrations. Adaptive optics (AO) offers a dynamic solution for reducing aberrations by detecting and correcting wavefront phase errors. Commonly, ophthalmic applications of AO employ Shack-Hartmann wavefront sensors (SH-WFS) to directly quantify wavefront errors [1–3]. These measurements guide the adjustment of deformable mirrors (DMs) or spatial light modulators (SLMs) to correct system aberrations [4,5]. However, the incorporation and calibration of SH-WFS can be complex. Furthermore, the efficacy of SH-WFS can be diminished in samples featuring multi-layered structures [4].

Sensorless adaptive optics (SAO) is an alternative to WFS-based AO that predicts wavefront aberrations based on the images directly. These image-based SAO methods could offer improvements to compactness, accessibility, and even the performance of AO systems [6]. The wavefront error can be reduced by maximizing the image sharpness, which can be represented by various pre-defined image metrics. The optimum-seeking scenario of SAO has been demonstrated using approaches such as stochastic parallel gradient descent (SPGD) [7], genetic algorithms [8], and simulated annealing [9]. While being accurate and suitable for SAO, these methods require lengthy optimization times and numerous iterations. Based on the orthogonality of Zernike modes and the assumption of their independent effects on the image metrics, a coordinate search method, such as Zernike mode hill climbing (ZMHC), iterates through the Zernike coefficient space to find the optimum image quality metric and correct the wavefront error [10–12]. Model-based approaches estimate the metric-coefficient curve, such as the Data-based Online Nonlinear Extremum-seeker (DONE) algorithm which addresses the wavefront error with fewer iterations than ZMHC and offers continuous coefficient predictions. DONE also tempers the impact of historical measurements and allows newer data to have a stronger influence on the model, offering a feature particularly beneficial in target movement or blinking scenarios [13–15]. Challenges remain despite these advancements, particularly regarding the time required for the iterative processes. Non-iterative approaches have also been reported. By exploiting the physical relationship between the mean square of the aberration gradient and the second moment of far-field intensity distribution, model-based methods reconstruct the aberrated wavefront by introducing perturbations on the DM under different modes [16,17]. Based on the understanding of the impact of the aberrated wavefront on pre-defined image quality metrics, model-based methods can estimate the wavefront error by applying bias disturbance signals to the wavefront control devices through a fixed number of steps [18–21]. The image data from OCT systems can be utilized for direct wavefront aberration extraction through digital or computational AO techniques [22–24]. While not iterative, DAO methods usually necessitate either a stationary sample or rapid OCT data collection to maintain phase stability.

Deep learning has emerged as a transformative force in SAO control, introducing novel approaches to wavefront estimation and correction. Most deep learning methods for SAO use aberrated images as the input and a Zernike coefficient decomposition of the wavefront as the output to form a direct mapping, without iterations to approach the optimal image quality. Innovative wavefront sensing techniques using convolutional neural networks (CNNs) have been demonstrated, effectively estimating Zernike coefficients from a single intensity image, marking a significant advancement in image-based wavefront sensing [25]. Deep neural networks (DNNs) have also been demonstrated to detect the wavefront distortion from varying atmospheric turbulence conditions directly from the intensity images, thereby avoiding time-consuming iterative processes [26]. A conformal convolutional neural network (CCNN) has been developed to boost performance by pre-processing circular features into rectangular ones through conformal mapping, reducing the number of convolutional filters and enabling more efficient feature recognition of PSF images [27]. ResNet has also been used as a control algorithm to replace the traditional control algorithm, enhancing the real-time performance of a free space optical communication (FSOC) system [28]. An EfficientNet-B0 CNN has been used for SAO, providing higher speed and accuracy under different turbulence intensities than the ordinary CNN and ResNet networks [29]. An extreme learning machine (ELM) has also been applied to SAO, demonstrating faster training speed than CNN while achieving a similar 87% accuracy [30]. Deep reinforcement learning algorithms have also been applied for SAO control. DDPG has been demonstrated in simulation with point spread function (PSF) images as the input for the actor-network, running 9 times faster than the SPGD algorithm [31]. An object-independent image-based wavefront sensing approach forms an innovative mapping between wavefront phase functions and object-independent data formed by the ratio between two images acquired with different amounts of defocus, achieving very high Zernike coefficient prediction accuracy at the level of 10^−4^ of the wavelength [32].

In this report, we focus on sensorless wavefront correction using a custom-developed confocal scanning laser ophthalmoscope (SLO) for preclinical (small animal) imaging and a metric input method. Although this method requires more image acquisitions than one-shot image-input DNNs, the metric-input method simplifies the complex image pattern changes caused by aberrations and attenuates the effects of noise and artifacts, offering a sample-independent and robust decision-making SAO process for live ocular imaging. The remaining challenge is to reduce the lengthy correction time associated with the iterative metric-based SAO methods.

Our previous work in a metric-input DDPG control for SAO demonstrated the feasibility of deep deterministic policy gradient (DDPG) for SAO with 6-7 times fewer input images than the ZMHC method in a multi-modal ocular imaging system [33]. The method in [33] required 2N+1 initial observations plus subsequent dynamic observations to determine the system aberration gradually. Furthermore, a significant limitation of the initial work was the number of modes (only five were demonstrated) that could be corrected due to training time and network complexity.

Here we present a Multi-Observation Single-Step DDPG (MOSS-DDPG) framework that requires only 2N+1 initial observations to predict the system aberration. The observation structure of MOSS-DDPG inherits the pipeline from model based methods, using positive and negative bias amplitudes of Zernike coefficients to disturb the wavefront and estimate the global maxima of image quality metric [18,20]. A potential obstacle for model based methods is the crosstalk between different modes’ effects on the image quality metric domain, which can confuse the decision-making process of the models. With an experience-based self-learning strategy, MOSS-DDPG learns from experiences of interactions with the environment to form an optimal policy that maps the bias observations to an optimal Zernike coefficient estimation. Here the model that governs the coefficient estimation is learned by MOSS-DDPG instead of a physical model based on the understanding of the relationship between image metric and modal coefficients. The self-learning process allows MOSS-DDPG to identify and accommodate potential modal crosstalk and system imperfections in its predictions, with a clear goal of maximizing the image quality metric during training. Long short-term memory (LSTM) networks were integrated into MOSS-DDPG’s actor-network and critic-network and significantly simplified the hyperparameter tuning process and expedited the training. Based on the single-step structure, an exploration-enhancing technique that generates multiple observation-action-reward pairs at each episode is used to increase the amount of training data, stabilizing and expediting the overall training process.

Based on *in silico* results, MOSS-DDPG enables a wavefront error prediction for the first five radial orders ( N=12 Zernike modes, excluding piston, tip, and tilt). Furthermore, after a brief transfer learning process on the real system to adapt to the exact *in situ* metric-coefficient space, MOSS-DDPG exhibits comparable *in situ* performance as the ZMHC method with the acquisition of only 2×12+1=25 images, making it more than 10 times faster. A model based parabolic maximization method using 2N+1 steps has been implemented and compared to MOSS-DDPG [20]. The results show that MOSS-DDPG can potentially address modal crosstalk and heterogeneous imperfections across optical systems.

## 2. Methods

### 2.1 Imaging system overview

**Fig. 1.**
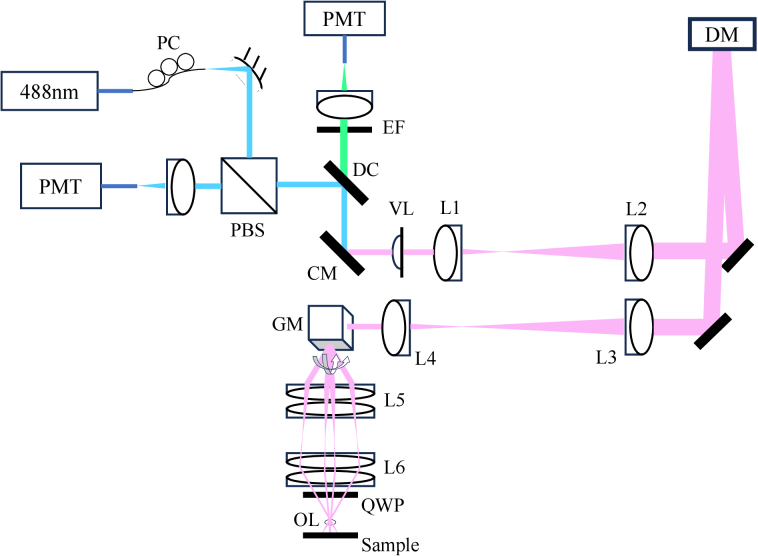
Optical layout of the SLO system. The blue represents the beam path of 488 nm light, the green represents the beam path of the fluorescence emission, and the red represents the beam path of the SLD light. The pink represents the co-aligned beam of the 488 nm and fluorescence light. Abbreviations: fiber coupler (FC), polarization beam splitter (PBS), dichroic mirror (DC), emission fiber (EF), cold mirror (CM), variable focus lens (VL), deformable mirror (DM), galvanometer-scanning mirrors (GM), quarter wave plate (QWP), photomultiplier tube (PMT), polarization controller (PC), objective lens (OL). $L_{n}$ represents lenses. Achromatic doublet lenses: L1=50 mm, L2=150 mm, L3=300 mm, L4=75 mm, L5=2x125 mm, L6=2x50 mm, OL=2.5 mm. Modified from [34].

The optical design of the system used to develop and test MOSS-DDPG is multi-modal, incorporating Optical Coherence Tomography (OCT), OCT-based Angiography (OCT-A), confocal Scanning Laser Ophthalmoscopy (SLO), and fluorescence detection [34]. SAO corrections are only performed on the SLO branch of the OCT-SLO system with a λ=488 nm laser. The schematic of the optical layout of the SLO branch is presented in Fig. 1. The imaging beams were relayed to the continuous membrane DM (DM69, Alpao, France) with a 10.5 mm aperture, and then to a mounted pair of Galvanometer-scanning Mirrors (GM, 6210H, Cambridge Technology Inc., MA, USA) with a clear aperture of 3.0 mm . Finally, the light was reduced to a beam diameter of 1.0 mm at the sample lens via the final optical relay.

The wavefront phase error of the system can be expressed by the superposition of a series of Zernike polynomials $Z_{i}(x,y)$ with corresponding Zernike coefficients $c_{i}$ :

(1) $$W(x,y)=\sum_{i}c_{i}Z_{i}(x,y),$$

where x and y are the coordinates of the wavefront plane and *i* is in the Noll index [35,36]. In the simulation, the Zernike coefficients are normalized to match the physical size of the coefficients in *μm* of the DM in the SLO system. The generalized pupil function *P* of the system is given by

(2) P(x,y)=A(x,y)exp⁡{−jW(x,y)},

where *A* is the amplitude transmission function of the pupil, e.g. 1 inside the pupil, 0 outside the pupil. The intensity point spread function (PSF) *h* is given by the magnitude squared of the Fourier transform of the pupil function:

(3) $$h={\left|F\{P(x,y)\}\right|}^{2}.$$

The double-pass and confocal nature of the system indicates a multiplication of both the illumination and collection PSFs. The incident light propagates through the system and arrives at the sample. Light from the sample is transmitted through the system in reverse and detected by the PMT. If the PSF of the illumination path is $h_{1}$ and the PSF of the detection path is $h_{2}$ , the effective PSF of the system $h_{s}$ is given by the multiplication of $h_{1}$ and $h_{2}$ , assuming a point detector [37]:

(4) $$h_{s}=h_{1}h_{2}.$$

However, a multi-mode fiber is used to receive the optical signal, which has a diameter of multiple times the Airy Disk diameter (ADD) and is therefore not point-like. To better emulate the real system’s intensity response to Zernike coefficients in simulation, we also considered the fiber’s effect on image formation. The effective PSF $h_{s}$ of the system considering the optical fiber’s propagation field *f* is the multiplication between $h_{1}$ and the fiber function *f* convolved with $h_{2}$ [38]:

(5) $$h_{s}=h_{1}(f⊛h_{2}).$$

To simplify the simulation while emulating the system’s behaviors, we use a unit circle with uniform values as the fiber’s intensity response and an adjustable diameter as the fiber’s size. Increasing the complexity of the simulation with complex fiber functions has diminishing returns because the network weights trained *in silico* can be easily modified to fit the real system using transfer learning.

With the definition of effective PSF, the fluorescence intensity signal received by the PMT is the convolution between the effective PSF and the sample’s fluorescence intensity profile $r_{f}$ [37]:

(6) $$I=h_{s}⊛r_{f}.$$

To evaluate the image quality, we use a sharpness-based image metric *m* :

(7) $$m=\sum_{p}I_{p}^{2}$$

which sums every pixel’s intensity squared in the image [39,40]. This metric effectively evaluates the image quality guiding the DDPG agent to find the Zernike coefficient solution to achieve the best image quality. Based on our successful *in silico* and *in situ* validation tests, the intensity-based metric effectively captures the influence of aberrations on the intensity of images and is image-independent for the specific settings of the SLO system.

### 2.2 MOSS-DDPG architecture

**Fig. 2.**
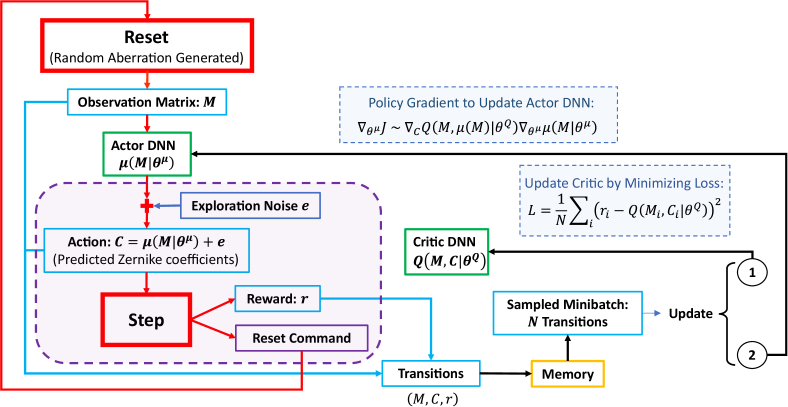
MOSS-DDPG structure for SAO. The red blocks and arrows represent the iteration of DDPG training episodes. The green blocks are the actor and critic DNNs. Light blue boxes represent the criteria for updating the actor and critic DNNs. Target networks are omitted as each episode consists of a single step without future rewards. The purple region surrounded by the dashed purple line represents the action evaluation process, where multiple noise profiles can be generated and added to the network prediction in one episode to enhance the model’s exploration of the environment.

A Deep Reinforcement Learning (DRL) approach was used for the SAO Zernike mode control. DRL combines conventional RL and Deep Neural Networks (DNNs), where DNNs act as the RL agent. The Deep Deterministic Policy Gradient (DDPG) algorithm is an extension of the Deep Q Network (DQN) method [41] using an Actor-Critic structure. DDPG is compatible with continuous domains of the SAO environment, where the states are the observation matrices of image metric values, and the actions are the Zernike coefficients. Figure 2 is a detailed MOSS-DDPG structure that inherits the traditional DDPG structure [42] and is tailored for the multi-observation single-step sensorless adaptive optics scenario.

Similar to the human response to stimuli, the DDPG agent observes the state of the environment and executes an action through the actor-network. In the context of SAO, the process involves acquiring an observation matrix from the aberrated system and predicting the Zernike coefficients that correct the wavefront error. It also has a user-defined reward strategy and a critic-network to evaluate the quality of the actions. During training, the actor-network keeps updating the policy toward improved accuracy in predicting wavefront errors based on the policy gradient.

The “reset” function is called at the start of the DDPG training episode. At this stage, the DDPG agent is given an artificial wavefront error formed by random Zernike coefficients. Subsequently, the agent constructs the observation matrix by introducing to the wavefront a series of coefficient combinations. These combinations include each mode’s coefficients set to both negative and positive values, along with a zero-offset setting for each mode. This approach generates 2N+1 different combinations and associated image metric values, where *N* represents the number of target modes.

**Fig. 3.**
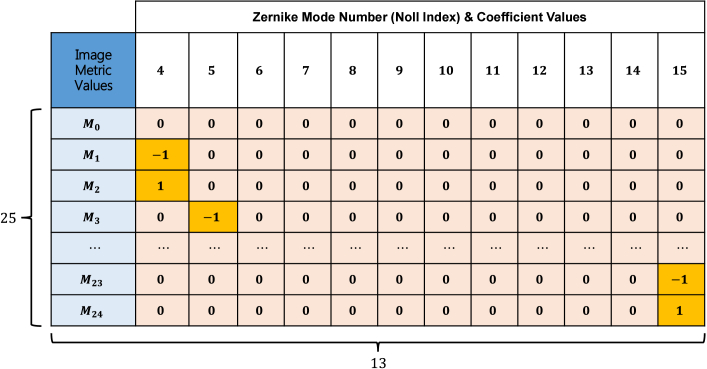
Observation Matrix for DDPG Actor-network.

The details of the observation matrix are shown in Fig. 3. The right 12 columns are the set of 2×12+1=25 observation types, and the leftmost column is the corresponding image metric values. To maintain a uniform value level for both the metric values and the coefficients in the observation matrix, the elements in the first column are normalized within the range of [0,1] as the portion of the largest image metric value:

(8) $$M_{n}=\frac{m_{n}}{\max\{m_{0},m_{1},\ldots ,m_{24}\}}.$$

For the right 12 columns, a set of signed numbers {−1,0,1} is used to represent the status of the observations. “0” represents the unobserved mode in the observation. "-1" and "1" represent the negative and positive observations of the mode, respectively.

The choice of observation values is flexible and can be an adjustable parameter for different system configurations. Although models trained *in silico* demonstrate similar effectiveness across various observation value settings, it is advisable to tune the value for *in situ* applications. Using a larger observation value moves the measurement further from the peak of the Gaussian-like metric-coefficient function curve, enhancing resilience to potential distortions caused by system imperfections. However, an excessively large observation value might significantly reduce image brightness, yielding image metric values that are markedly low and difficult to distinguish. In this study, we uniformly apply an observation value of 0.5 *μm* across all modes.

After reset, the actor-network takes the observation matrix and outputs a series of predicted Zernike coefficients based on the current network weights. A zero-mean Gaussian exploration noise is added to the actor’s output, enhancing the exploration of the metric-coefficient space. During training, the standard deviation (SD) of the noise is gradually reduced (annealed) as the actor-network’s prediction accuracy improves.

The “step” function then examines the quality of the actor-network’s prediction by applying the predicted coefficients to the DM, acquiring the image, and calculating its metric value. The reward is calculated by dividing the current metric value by the maximum metric value, the latter determined when all Zernike coefficients are set to zero. This reward mechanism guides the agent in optimizing the Zernike coefficients to effectively cancel out the system’s aberrations. Under *in situ* scenarios where the maximum metric value is not precisely known, a baseline image metric value acquired from a wavefront close to flatness will be used. Besides the reward, the “step” function generates a Boolean reset command, which is always “True” since the agent only performs one-step prediction before the environment reset.

The single-step structure of MOSS-DDPG maps each observation and action directly to an immediate reward. This design feature allows for the generation of multiple noise profiles within each episode, each of which can be individually added to the actor network’s predictions. These actions, each with a different noise addition, are processed by the ’step’ function to generate the corresponding rewards, tailoring the training to diverse scenarios. This strategy significantly enhances the agent’s exploration of the metric-coefficient space. The bottleneck for shortening MOSS-DDPG training time is the amount of convolutions between the target image and the PSFs. Each episode’s observation matrix inherently requires 25 convolutions. Introducing additional single convolutions per episode will marginally extend the training time, yet substantially increase the volume of training data. With enhanced exploration, the model achieves more efficient training and requires fewer episodes to reach proficiency.

Observations, actions, and rewards from each episode are stored as transition pairs in the memory. Before initiating the next episode, the agent samples a user-defined number of transition pairs from the memory. These pairs are then used to update the actor and critic networks, as depicted in Fig. 2. Conventional DDPG employs target networks for both the actor and the critic to provide a stable target during the learning updates. Since each episode of MOSS-DDPG has an immediate reward based on the action, the concept of future rewards, which target networks help to stabilize by providing a moving target, is not applicable in this scenario.

### 2.3 LSTM for DDPG DNN

Our previous work realized a 5-mode DDPG SAO model with a fully connected actor-network [33]. However, the dense networks are susceptible to hyperparameter tuning and converge slowly. Experimentally training the networks was prone to failure because of the inadequate choices of hyperparameters.

The observation matrix of MOSS-DDPG is characterized by sequential sets of Zernike coefficients alongside their corresponding image metric values. Long Short-Term Memory (LSTM) networks are particularly well-suited for learning from these samples, leveraging their capability to process data with sequential dependencies effectively [43]. LSTM networks can potentially minimize the ’invalid’ interactions that may arise in fully connected networks, which often occur due to the treatment of observation matrix elements as independent, unrelated inputs.

## 3. Results

### 3.1 Model training

#### 3.1.1 Zernike coefficient correction limit & random aberration generation

Before training, it is crucial to establish an effective Zernike coefficient correction limit that will generate a sufficient range of random aberrations for comprehensive training and validation. The confocal SLO system’s image metric response to Zernike coefficients is sensitive. Consequently, a correction limit that is too large results in weak image signals from which effective information cannot be observed, particularly when many of the 12 coefficients are near this limit. Therefore, we set an initial limit for Zernike coefficients to 0.15 *μm* for all 12 modes during training. Each coefficient is uniformly distributed within the range of [−0.15,0.15] μm . Considering the shorter laser wavelength λ=0.488 μm and the smaller beam width of 1 *mm* at the mouse eye in our SLO system compared to the settings used in measuring mouse eye aberrations [44], the limit of 0.15 *μm* is sufficiently large. This ensures robust coverage of the mouse eye aberration spectrum, providing ample room for accurate analysis and interpretation. Furthermore, we have demonstrated that the model can extrapolate effectively to Zernike coefficients with a larger limit of 0.2 *μm* using the learned policy, thereby broadening the model’s effective prediction range.

To emulate the real application scenarios where residual higher-order Zernike mode components are present, random wavefront distortions formed by Zernike modes in the 6th and 7th radial order are also applied to the simulated wavefront. As the mouse eye aberration consists of mostly lower-order mode components [44], the higher-order mode components are simulated as noise with small amplitudes. For simplicity, the Zernike coefficient for all modes in the 6th and 7th order follows the Gaussian distribution with a standard deviation of 0.01 *μm*. A limit of [−0.025,0.025] μm is also applied. The higher-order mode noise helps emulate *in situ* imaging sessions and can contribute to a smoother *in situ* transfer learning process with increased robustness.

#### 3.1.2 Simulation environment & DNN architectures

The MOSS-DDPG training and testing is conducted within a Python-based simulation environment, utilizing OpenAI Gym to build the DRL environment structure, PyTorch for the network architectures, and CuPy to accelerate the convolutions. The details of the DNN structures and MOSS-DDPG training hyperparameters are exhibited in the Supplement 1.

#### 3.1.3 Training process

**Fig. 4.**
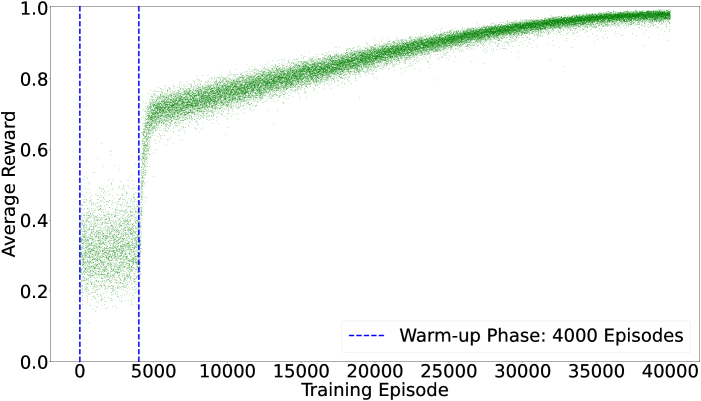
MOSS-DDPG training record. Each green dot represents the average reward achieved by 15 actions added with random noise profiles in each episode. The entire training process took 3 hours on a desktop computer with an Intel i5-13600K CPU and an Nvidia RTX 4080 GPU.

In each training episode, 15 random noise profiles are added to the action to acquire corresponding rewards. This exploration-enhancing technique significantly increases the total volume of training data by 15 times, while only requiring 40% more time for image convolutions. In contrast to previous work, where 200,000 episodes were used to train a 5-mode model [33], MOSS-DDPG requires only 40,000 episodes to effectively learn an optimal policy for correcting 12 modes. The exploration noise is a zero-mean Gaussian noise with an annealing SD as the training progresses. We have applied a warm-up phase to the training process. During this phase, the networks remain static, exploring the environment solely through the introduction of random noise. The warm-up phase accumulates sufficient data to stabilize the training process before network updates commence. Figure 4 is a scatter plot exhibiting the trend of the average reward in each training episode.

Each green dot in Fig. 4 represents the average reward of the corresponding training episode. Notably, the reward increases sharply upon completion of the warm-up phase. With the exploration-enhancing technique, 4,000×15=60,000 training samples have already been generated at the end of the warm-up phase. Therefore, with a large volume of data, the actor-network and critic-network are able to update both swiftly and steadily. In the early stages of training, the agent explores the Zernike coefficient space using a high SD for the noise. The rewards are small in value and sparse in their distribution, resulting from both the immature policy and the large noise SD. As the noise SD decreases, MOSS-DDPG gradually updates the network weights based on past experiences, enhancing its predictions and leading to the optimal policy by the end of training when the noise SD reaches zero.

### 3.2. *In-silico* performance evaluation

The effectiveness of MOSS-DDPG in correcting wavefront errors can be quantitatively assessed through various metrics, such as the root-mean-square (RMS) residual error of the wavefront, image metric relative to the image metric acquired without aberrations, and the amplitude of the Zernike coefficient error. Given that the aberrations in our simulations are synthetically generated and precisely known, we employ a comparative analysis of Zernike coefficients between the model’s predictions and the actual aberrations as our primary metric for performance evaluation. This comparison yields the residual errors in Zernike coefficients, which form the RMS wavefront error ${\text{RMS}}_{\text{WFE}}$ , defined by:

(9) $${\text{RMS}}_{\text{WFE}}=\sqrt{\sum_{i=4}^{15}{\left(c_{e}^{i}\right)}^{2}}$$

where $c_{e}^{i}$ represents the coefficient prediction error for the i-th mode of the Noll index. To statistically evaluate the performance of MOSS-DDPG’s prediction errors, we will average the ${\text{RMS}}_{\text{WFE}}$ across test samples. To clarify, since MOSS-DDPG corrects for only the first 12 Zernike modes (excluding piston, tip, and tilt), the performance evaluation metric ${\text{RMS}}_{\text{WFE}}$ is only evaluating the accuracy for the first 12 modes. The contribution of residual higher-order mode noise on the wavefront error is not considered, as opposed to the conventional RMS wavefront error definition. The overall RMS wavefront error considering higher order mode noise will be higher than the results presented below.

To make a comparison to the model based methods that also use observation biases for each mode to estimate the modal coefficients, a parabolic maximization method based using the same 2N+1 observations as MOSS-DDPG has also been tested. The coefficients $c_{n}^{corr}$ are determined through parabolic maximization as [45]:

(10) $$c_{n}^{corr}=\frac{b(M_{n}^{+}-M_{n}^{-})}{2M_{n}^{+}-4M_{0}+2M_{n}^{-}}$$

where *n* is the Zernike mode index, *b* is the observation bias value, $M_{0}$ is the image metric acquired without applying observation bias, and $M_{n}^{+}$ and $M_{n}^{-}$ values represent the observed image metric values for the n-th mode with positive and negative observation biases, respectively. Based on experimental tests, the observation bias value has a large impact on the performance of the parabolic maximization method. A 0.2 *μm* observation bias value is used for all modes to achieve the best performance.

The training environment using uniformly distributed random coefficients for the wavefront error is initially designed for the model to experience a broad range of aberrations. This uniform distribution may result in scenarios where several mode coefficients approach the limit of 0.15 *μm*, potentially decreasing image intensity and prediction accuracy. However, these extreme cases occur infrequently in practical scenarios. To mitigate cases where many coefficients approach the limit, we adopted a Gaussian distribution with an SD set at 0.4 times the limit. This distribution ensures that approximately 80% of the coefficients remain within half the limit, and 98.76% do not exceed the limit. With the Gaussian distribution, it is less likely that random Zernike coefficients of different modes will simultaneously reach the limit, though the possibility remains for individual coefficients.

We present three distribution configurations to statistically examine the performance of MOSS-DDPG:

A: Uniformly distributed random Zernike coefficients within the [−0.15,0.15] μm limit, representing the random wavefront error that MOSS-DDPG experienced during training.
B: Zero-mean Gaussian-distributed random Zernike coefficients with a standard deviation of 0.06 *μm*, constrained within the [−0.15,0.15] μm limit. This configuration minimizes the likelihood of multiple coefficients reaching the limit simultaneously.
C: The 12 Zernike modes were categorized into three groups based on their radial order, with decreasing limits for each group; the details are provided in Table 1. Zernike coefficients typically decrease as the mode index increases and most aberrations predominantly originate from lower-order modes, especially in scenarios involving mouse eye aberrations [44]. This configuration also allows us to assess the model’s extrapolation ability on unseen wavefront errors by setting the coefficient limits to [−0.2,0.2] μm .

**Table 1.** Configuration C: Gaussian-distributed random Zernike coefficients with decreasing standard deviations

Radial Order	Zernike Modes	Limits	Gaussian Distribution SD
3rd	4,5,6	[-0.2,0.2] *μm*	0.08 *μm*
4th	7,8,9,10	[-0.2,0.2] *μm*	0.06 *μm*
5th	11,12,13,14,15	[-0.2,0.2] *μm*	0.04 *μm*

**Fig. 5.**
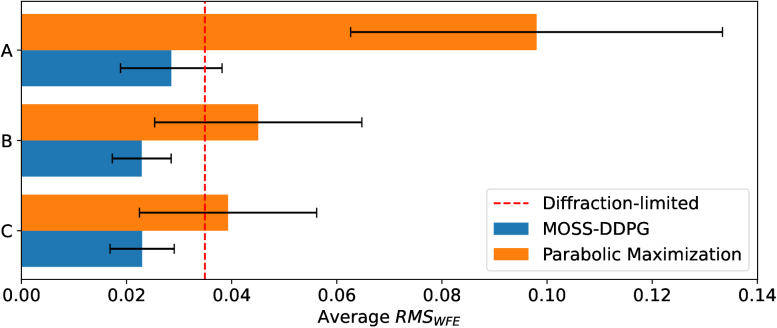
Average RMS wavefront errors after corrections of aberrations from MOSS-DDPG and parabolic maximization under different random Zernike coefficient distribution configurations. Configuration A: 0.15 *μm*-limit uniform distribution. Configuration B: 0.15 *μm*-limit Gaussian distribution with 0.06 *μm* standard deviation. Configuration C: 0.2 *μm*-limit Gaussian distribution with decreasing standard deviation as in Table 1. Orange bars: parabolic maximization correction results. Blue bars: MOSS-DDPG correction results. Average ${\text{RMS}}_{\text{WFE}}$ values: 0.098 (A, orange), 0.029 (A, blue), 0.045 (B, orange), 0.023 (B, blue), 0.039 (C, orange), 0.023 (C, blue). The T bars represent the standard deviation values of ${\text{RMS}}_{\text{WFE}}$ of 1000 tests: 0.035 (A, orange), 0.009 (A, blue), 0.019(B, orange), 0.006 (B, blue), 0.017 (C, orange), 0.006 (C, blue). The red dashed line represents the the Maréchal criterion λ/14=0.0349 μm for diffraction-limited ${\text{RMS}}_{\text{WFE}}$ .

We have conducted 1,000 tests to examine the performances of MOSS-DDPG and the parabolic maximization method under each of the three configurations. MOSS-DDPG and the parabolic maximization method experience the same random aberrations, and the prediction results of both are recorded for fair comparisons. Figure 5 presents the average ${\text{RMS}}_{\text{WFE}}$ values after the correction of MOSS-DDPG and parabolic maximization.

From the results of 1000 tests, MOSS-DDPG exhibits better performance in predicting random aberrations under all three configurations. The parabolic maximization method performance drops drastically under configuration A when the aberrations are uniformly distributed across 0.15 *μm*. Compared to the Gaussian-distributed random aberration, configuration A generates larger modal crosstalk when many of the random coefficients reach the limit. For configuration B and configuration C, parabolic maximization performs reasonably but does not reach the same level of accuracy as MOSS-DDPG.

For MOSS-DDPG:

1. The average ${\text{RMS}}_{\text{WFE}}$ of 0.029 *μm* of configuration A (upper blue bar) is the largest among the three configurations since it has a higher possibility of generating large wavefront errors with multiple coefficients simultaneously approaching the 0.15 *μm* limit. Nevertheless, the average ${\text{RMS}}_{\text{WFE}}$ remains below the Maréchal criterion after correction. This outcome demonstrates that MOSS-DDPG has effectively explored the environment and learned an optimal policy to optimize image quality.
2. The middle blue bar in Fig. 5 represents the average ${\text{RMS}}_{\text{WFE}}$ of 0.023 *μm* after MOSS-DDPG’s corrections with random coefficients generated from configuration B. Compared to A, MOSS-DDPG performs better under B with a smaller average ${\text{RMS}}_{\text{WFE}}$ and a smaller SD. This is due to the reduced possibility of multiple coefficients simultaneously approaching the limit.
3. The lower blue bar in Fig. 5 represents configuration C, which emulates the mouse eye aberrations. The average ${\text{RMS}}_{\text{WFE}}$ value of 0.023 *μm* is also below the Maréchal criterion. The small SD of 0.006 *μm* positions the Maréchal criterion at about 2 SD above the average, indicating that 97.7% of the MOSS-DDPG correction results under configuration C meets the Maréchal criterion, assuming the ${\text{RMS}}_{\text{WFE}}$ has a Gaussian distribution. Additionally, the success of MOSS-DDPG in predicting extrapolated coefficients to 0.2 *μm* proves that MOSS-DDPG captures the nature of the metric-coefficient space instead of just forming a look-up table between observed metrics and coefficients.

We have also tested the independence of MOSS-DDPG from the image contents by using a wide variety of images: images of ganglion cells that show detailed neural structures and lens tissue images with both wide and thin fibrous lines acquired in real systems. The accuracy and stability of MOSS-DDPG and the parabolic maximization method we observed were consistent across all these different types of images. This demonstrates that the sharpness-based image metric captures the image quality metric response of a confocal microscope, does not depend on the details of the images, and can be applied effectively for various samples.

### 3.3 *In situ* validation

The *in situ* validation utilized artificial wavefront aberrations formed by randomly generated Zernike coefficients. These aberration patterns were translated into actuator controls and applied to the deformable mirror (DM). Unlike the perfect image formation environment in simulation, challenges exist for the implementation and evaluation of MOSS-DDPG in real systems. Rather than directly comparing MOSS-DDPG’s predicted coefficients with the unknown ground truth, we compared the image metrics after aberration correction from MOSS-DDPG to a benchmark that is close to the optimum that we established by employing the ZMHC coordinate search method. While the DONE method demonstrates faster convergence and potentially better accuracy than ZMHC, we opted for the simplicity of implementation and reliability of ZMHC. Post-correction image metric values by MOSS-DDPG were then compared to this benchmark. In each ZMHC round, 11 uniformly distributed coefficient values, spanning from the negative to the positive limit, were applied to the DM to identify optimal coefficients. The coefficient limit decreases across rounds: ±0.2 μm in the first, ±0.1 μm in the second, and ±0.05 μm in the final round. The resolution of ZMHC for each coefficient is 0.01 *μm*, determined by the step size of the last round. This specific strategy of coordinate search requires 132 images per round and 396 images in total.

#### 3.3.1 Transfer learning

In our specific application scenario (SAO), while the real system exhibits a metric-coefficient response similar to the simulation, minor alignment imperfections in the real system and approximations in the simulation can degrade the performance of a directly implemented *in-silico* MOSS-DDPG. We have demonstrated that a very brief transfer learning process on the real system with pre-learned weights in simulation can vastly improve the *in situ* performance of MOSS-DDPG.

By definition, transfer learning aims at improving the performance of the model on target domains by transferring the knowledge contained in different but related source domains [46]. In this section, we first present a brief transfer learning process to better fit the MOSS-DDPG trained *in-silico* to the real system. We subsequently evaluate the performance of both the directly applied and the transfer learning-enhanced versions of MOSS-DDPG, by comparing the corrected image metrics to the benchmark image metrics established by ZMHC. Finally, we present and compare the decision-making processes of MOSS-DDPG and the ZMHC method.

Before training, the transfer learning model inherits the weights from both the actor-network and the critic-network of the *in-silico* MOSS-DDPG. The generation of random aberrations follows the decreasing-limit strategy outlined in Table 1. The reward for training is defined as the relative image metric, which is compared to the benchmark image metric established by the ZMHC method.

**Fig. 6.**
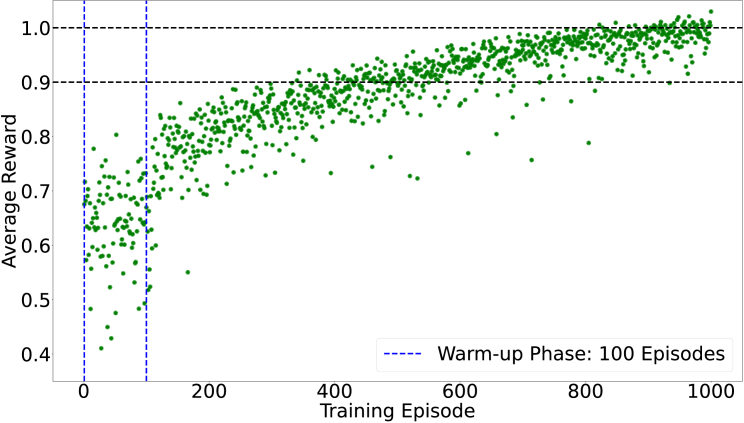
Transfer learning process of MOSS-DDPG. The transfer learning takes 1,000 episodes. Green dots represent the image metric of MOSS-DDPG’s prediction relative to the ZMHC. The entire training process took 45 minutes on a desktop computer with an Intel i9-10900X CPU and an Nvidia RTX 3060 GPU with 12 GB memory.

Figure 6 exhibits a transfer learning session. The transfer learning involves 1,000 episodes, which constitutes only 2.5% of the episodes used in the simulation. 7 actions with random noise are generated at each training episode for enhanced exploration. At the start, exploration noise is added to MOSS-DDPG’s predictions to help it explore the real system environment. As training progresses, MOSS-DDPG’s image metric gradually converges to the ZMHC benchmark. Occasionally, the image metric values following MOSS-DDPG’s predictions exceed the ZMHC benchmark, indicating potential fluctuations in detected intensity between the two processes or superior solutions identified by MOSS-DDPG. Given that ZMHC has limited resolution and does not accommodate potential mode interactions resulting from system imperfections, MOSS-DDPG can indeed identify more effective solutions through its continuous predictions of Zernike coefficients.

#### 3.3.2 *In situ* performance evaluation

**Fig. 7.**
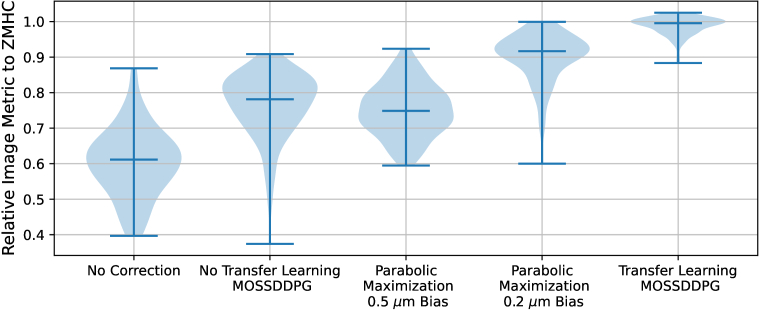
Violin plot comparing relative image metrics to ZMHC across different correction techniques, including No Correction, No Transfer Learning MOSS-DDPG, Parabolic Maximization with 0.5 μm and 0.2 μm Bias, and Transfer Learning MOSS-DDPG. The blue bars indicate the maximum, minimum, and median values for each group. “Transfer Learning MOSS-DDPG” shows the highest and most consistent performance, while “No Correction” is the least as it represents the image metrics from the aberrated wavefront patterns before correction.

To examine the *in situ* performances of MOSS-DDPG both before and after transfer learning and compare its performance with parabolic maximization, we have generated 100 random aberrations that follow the limit strategy in Table 1. Figure 7 displays the test results of image metrics (relative to ZMHC) following wavefront error corrections by MOSS-DDPG and parabolic maximization, both before and after transfer learning. Two observation bias values have been tested for parabolic maximization: 0.2 *μm* and 0.5 *μm*; based on the experiments, the 0.2 *μm* observation bias value performs better for the specific optical system and amount of aberration. The best performance was recorded using MOSS-DDPG with transfer learning.

With the image metric values reduced to approximately a median of 61% of the benchmark due to random aberrations, the direct application of *in silico* MOSS-DDPG demonstrates a significant improvement, reaching a median of 78% of the benchmark. After transfer learning, the refined MOSS-DDPG model further improves the metric values of aberrated images to a median of 99% of the benchmark. MOSS-DDPG enhanced by transfer learning not only achieves performance comparable to that of ZMHC but also demonstrates a marked improvement in the stability and accuracy of its predictions over the directly implemented *in silico* MOSS-DDPG.

Two parabolic maximization tests with different bias values exhibit distinct performances. 0.5 *μm* bias parabolic maximization achieves a median of 75% of the benchmark metric while 0.2 *μm* bias parabolic maximization improves the median to 91%.

All tested models exhibit the ability to improve the image quality metrics by predicting the Zernike coefficients that form the random aberration patterns. The MOSS-DDPG model after transfer learning stands out for its high and consistent performance. Parabolic maximization with 0.2 μm bias also demonstrates high-level accuracy, however, the distribution of image metrics after corrections is not as narrow, indicating some variability in performance, which can be caused by modal crosstalk.

Due to the non-uniform flatness of the sample, variations in focal planes and aberrations are present throughout the entire field of view. To assess the model’s robustness to the artifacts and its adaptability to images with diverse spatial frequency distributions, the sample underwent both vertical and horizontal shifts. Under each movement, MOSS-DDPG resets its predictions and corrects various aberrations with different image contents. The images corrected by MOSS-DDPG consistently achieve over 95% of the image metric attained by ZMHC on average, indicating their sharpness is comparable to ZMHC results with only minor further refinements.

#### 3.3.3 Decision-making process visualization

**Fig. 8.**
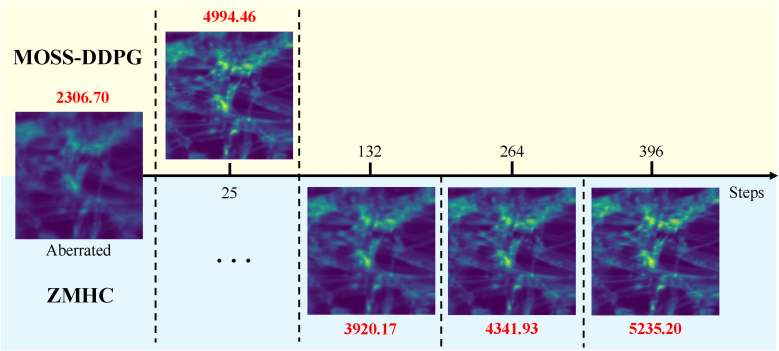
*In situ* correction process comparison between MOSS-DDPG & ZMHC. The x-axis is the number of image acquisitions (steps) experienced by the two models. The upper half with a yellow background is MOSS-DDPG and the lower half with a blue background is the ZMHC. The images’ metrics associated with correction steps are noted in red color.

The sample being imaged is a part of a lens tissue covered by fluorescent ink. The decision-making process of MOSS-DDPG is compared to the ZMHC method of three rounds of correction. The prediction process for both the MOSS-DDPG model and the ZMHC model is shown in Fig. 8.

Before the aberration correction process, the aberrated image has an initial metric value of 2,307. After the acquisition of 25 images, MOSS-DDPG predicts Zernike coefficients for 12 modes to correct the system aberration, improving the image quality metric by 117% . Being exhaustive and slow with coordinate search, the ZMHC takes 132 steps in the first round to make its first prediction, effectively addressing the major aberrations but lacking the finer adjustments. In its second iteration, ZMHC achieves a reward comparable to that of MOSS-DDPG but takes 264 steps, 10 times more than that of MOSS-DDPG. After the fine-tuning of Zernike coefficients in the last round, ZMHC reaches the highest image metric value among all and outperforms MOSS-DDPG by 4.8% .

**Fig. 9.**
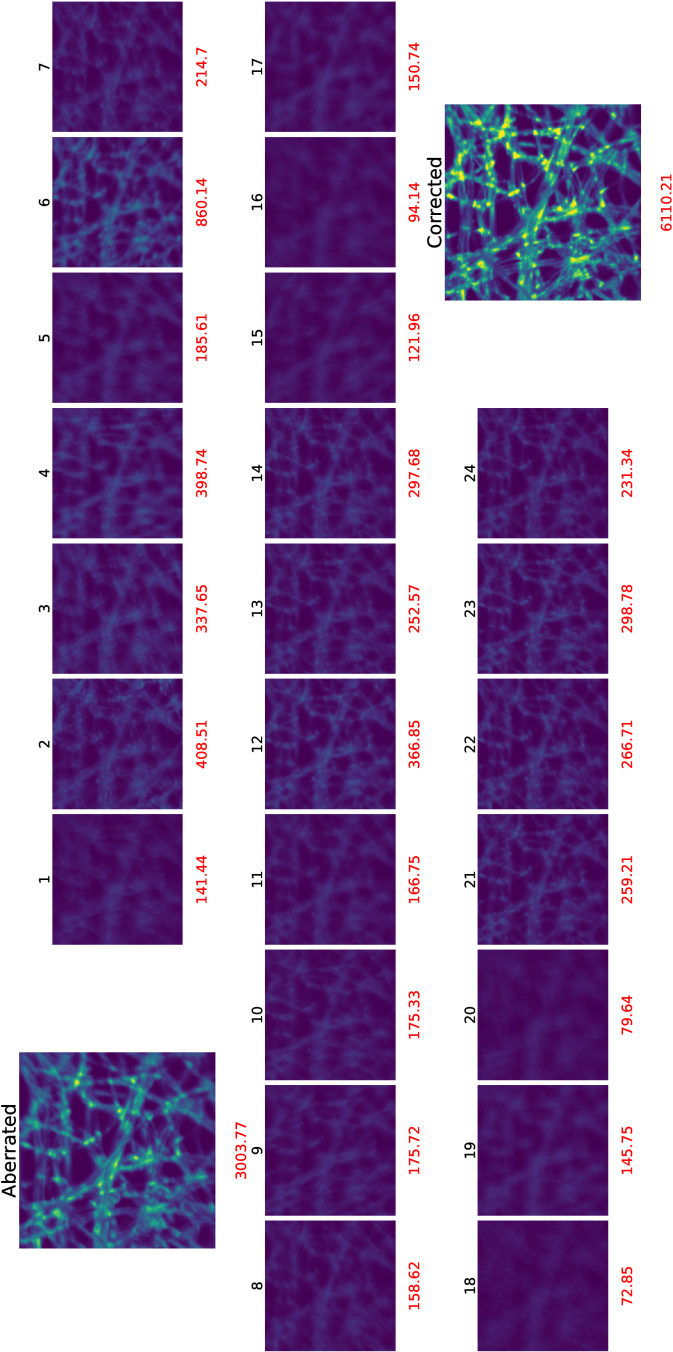
Decision-making progress of MOSS-DDPG. The black numbers are the sequence of the image metric value acquisition to form the observation matrix. The red numbers are the image metric values for each image. The corrected image improves the image metric by over 100%.

Figure 9 is an example of the decision-making process of MOSS-DDPG in correcting a randomly generated aberration with a different location of the lens tissue and a larger field of view (FOV) than Fig. 8. The aberrated image before correction has a metric value of 3,004 and is blurry. The corrected image exhibits much more details than the aberrated image with a metric value of 6,110, improving the metric by over 100%.

## 4. Discussion

By using the Deep Deterministic Policy Gradient with a single-step setting, we have realized a fast and accurate optimization of *N* Zernike coefficients with an observation matrix of 2N+1 image quality metric values, within a Zernike coefficient range of [-0.2, 0.2] *μm*. Using an exploration-enhancing technique that generates multiple noise profiles for each training episode significantly increases the overall training speed. Integrating Long Short-term Memory networks also stabilizes the training process and improves the training speed, making possible a 12-mode wavefront error correction within an efficient Zernike coefficient limit that effectively covers the Zernike coefficient range of mouse eye aberration. MOSS-DDPG can be trained completely *in silico* and applied to *in situ* scenarios with a brief transfer learning process. This ensures fast model training sessions with modest computational power and broad adaptability to *in situ* imperfections like non-common path aberrations [47].

The demonstration of MOSS-DDPG’s potential of addressing the modal crosstalk and system imperfections involves comparisons with a 2N+1 model based parabolic maximization method. We recognize that there are other model based methods with higher complexity and better performance [21]. Optimum modal construction instead of the more widely used Zernike modes can be developed to improve the robustness of model based correction methods [40,45]. In addition, only uniform bias values across modes were tested for the parabolic maximization method, and non-uniform bias values can be explored to achieve better parabolic maximization performance. The simple implementation of a 2N+1 parabolic maximization method might overclaim the performance of MOSS-DDPG. Nevertheless, from the correction results, the higher instability and lower accuracy of the parabolic maximization method compared to MOSS-DDPG demonstrates modal crosstalk and potential system imperfections that hamper the parabolic property of the metric-coefficient curve. With a learning-based strategy, MOSS-DDPG has the potential to overcome these obstacles.

Direct application of the *in silico* trained MOSS-DDPG does not produce the most desirable output on a real system but can be improved by brief transfer learning processes. A reason for the difference in performance is that approximations were made in simulating the fiber function as a simple unit circle, and the circle diameter was experimentally determined in the simulation. This approximation might form a slightly different relationship between image metric values and Zernike coefficients in the simulation versus the real system. Other system imperfections, including the DM actuator accuracy, laser power stability, non-common path aberrations, and the galvanometer scanners can also affect the *in situ* metric-coefficient relationship that is challenging to simulate. These problems are commonly encountered for SAO models that are trained *in silico* and applied to *in situ* scenarios. An advantage of MOSS-DDPG is that it can quickly adapt to a new environment with transfer learning. Nevertheless, it is suggested that careful alignment and reduction of non-common path aberrations in the system are performed before applying MOSS-DDPG to ensure smooth and swift transfer learning processes and robust model predictions.

Although MOSS-DDPG’s policy self-learning process is iterative, once trained, it makes predictions in practical applications with a fixed number of image acquisitions without further iterations, supported by *in situ* experimental validations. Focusing more on the speed while maintaining reasonable accuracy, MOSS-DDPG distinguishes itself from fast iterative model based methods, such as DONE, which generally require a larger number of iterative steps (60-250) even for a modest number of modes (3-7) for every new aberration scenario [13,15]. Nevertheless, the iterative methods may eventually achieve better results after a large number of iterations. While MOSS-DDPG exhibits comparable performance to ZMHC with a single-shot prediction, it can also run iteratively to acquire multiple observation matrices, where each of the observation matrices is based on the previous corrected wavefront, further improving the prediction accuracy while maintaining the speed advantage.

MOSS-DDPG exhibits a high speed of operation, which significantly mitigates the impact of potential perturbations such as the movement or blinking of the sample during the optimization process. Its rapid processing capability ensures that even in the face of such disturbances, the likelihood of their affecting the optimization outcome is greatly reduced. Notably, even SAO methods capable of continuous update and optimization cannot bypass the theoretical limit of steps required when facing new aberrations. A straightforward reset of MOSS-DDPG in instances where aberrations change during live imaging maintains fast and high-quality optimization in dynamic aberration imaging environments.

MOSS-DDPG’s ability to make wavefront error predictions for 12 Zernike modes in only 25 steps makes it highly effective for live aberration correction. Future work can further improve the model’s prediction accuracy, training efficiency, coefficient range, Zernike mode range, and the stability of correction. More modes can be implemented for MOSS-DDPG as a trade-off between correction speed and correction accuracy. With more modes for correction, the negative influence of uncorrectable higher-order mode noise will be further mitigated. Other image quality metrics (such as the Strehl ratio [48] or encircled energy [49]) that provide a differentiable objective function for MOSS-DDPG to optimize can be explored. Expanding the use of alternative metrics beyond image sharpness would enhance the applicability of MOSS-DDPG to a broader range of adaptive optics scenarios. Applications of the MOSS-DDPG framework to other imaging systems with different image formation physics are also interesting areas of exploration.

## 5. Conclusion

This work demonstrated that the implementation of a multi-observation single-step deep deterministic policy gradient framework with LSTM actor and critic networks on a confocal scanning laser ophthalmoscope can effectively correct aberrations of 12 Zernike modes (the first 5 radial orders excluding piston, tip, and tilt). The correction process requires only 2×12+1=25 image acquisitions, representing the fewest steps possible for image metric-based SAO optimizations. The model can be trained *in silico* at first and then applied to *in situ* scenarios with brief transfer learning processes, significantly accelerating the overall training process compared to purely *in situ* training. With over 10 times faster speed than the coordinate search ZMHC method, MOSS-DDPG exhibits comparable performances for *in situ* wavefront error correction tests.

## Supplemental information

10.6084/m9.figshare.26252324 Supplement 1 Supplemental document https://doi.org/10.6084/m9.figshare.26252324

## Data Availability

The source code for MOSS-DDPG construction and performance evaluation is openly accessible at GitHub [50].

## Supplemental document

See Supplement 1 for supporting content.
