# Supplementary material for: Image metric-based multi-observation single-step deep deterministic policy gradient for sensorless adaptive optics
Source: Biomed Opt Express. 2024 Jul 23;15(8):4795–814. doi: 10.1364/BOE.528579 (PMC11427189; doi:10.1364/BOE.528579)
Supplement: Supplementary file 1 [file boe-15-8-4795-s001.pdf]

Supplemental Document

Biomedical Optics EXPRESS

Image metric-based multi-observation single-step deep deterministic policy gradient for sensorless adaptive optics: supplement

GUOZHENG XU,[1,*,†] THOMAS J. SMART,[2,†] EDUARD DURECH,[3] AND MARINKO V. SARUNIC[1,2,3]

[1] *Department of Medical Physics and Biomedical Engineering, University College London, London WC1E 6BT, United Kingdom*
[2] *Institute of Ophthalmology, University College London, London WC1E 6BT, United Kingdom*
[3] *School of Engineering Science, Simon Fraser University, Burnaby BC V5A 1S6, Canada*
[†] *contributed equally*
[*] *guozheng.xu.23@ucl.ac.uk*

This supplement published with Optica Publishing Group on 23 July 2024 by The Authors under the terms of the Creative Commons Attribution 4.0 License in the format provided by the authors and unedited. Further distribution of this work must maintain attribution to the author(s) and the published article's title, journal citation, and DOI.

Supplement DOI: https://doi.org/10.6084/m9.figshare.26252324

Parent Article DOI: https://doi.org/10.1364/BOE.528579

Image metric-based multi-observation single-step deep deterministic policy gradient for sensorless adaptive optics: supplemental material

GUOZHENG XU,[1,*,†] THOMAS J. SMART,[2,†] EDUARD DURECH,[3] AND MARINKO V. SARUNIC[1,2,3]

[1] *Department of Medical Physics and Biomedical Engineering, University College London, London, WC1E 6BT, United Kingdom*
[2] *Institute of Ophthalmology, University College London, London, WC1E 6BT, United Kingdom*
[3] *School of Engineering Science, Simon Fraser University, Burnaby, BC V5A 1S6, Canada*
[†] *contributed equally*
[*] *guozheng.xu.23@ucl.ac.uk*

1. Deep neural network structures for DDPG actor and critic networks

The Multi-Observation Single-Step Deep Deterministic Policy Gradient (MOSS-DDPG) training and testing is conducted within a Python-based simulation environment, utilizing OpenAI Gym and Keras-RL source code to build the deep reinforcement learning (DRL) environment structure, PyTorch for the network architectures, and CuPy to accelerate the convolutions. The details of the DNN structures and MOSS-DDPG training hyperparameters are listed in the following tables. Table S1 describes the structure of the critic-network, and Tables S2 shows the structure of the actor-network.

Table S1. Critic-network structure

Layer Input	Dimension	Activation	Layer Output
**Observation Matrix**	128 LSTM Units	tanh	Final Hidden State
Final Hidden State	12	tanh	Processed Observation
Processed Observation + **Action**	500	ReLU	Linear Output
Linear Input	1000	ReLU	Linear Output
Linear Input	1000	ReLU	Linear Output
Linear Input	500	ReLU	Linear Output
Linear Input	300	ReLU	Linear Output
Linear Input	200	ReLU	Linear Output
Linear Input	1	Linear	**Reward**

[a] Except for the LSTM layer, all other layers are fully connected.
[b] The 2-dimensional observation matrix is first flattened to 1D by a structure similar to the actor-network and is then concatenated with the 1D action. The concatenated structure is the input for the following fully connected layers.

Table S2. Actor-network structure

Layer Input	Layer Dimension	Activation	Layer Output
**Observation Matrix**	128 LSTM Units	tanh	LSTM Final Hidden State
LSTM Final Hidden State	12	$0.3 \times \tanh$	**Action**

As detailed in Table S2, the actor-network is a two-layer neural network designed to process observation matrices and output actions. The first layer, an LSTM [1], handles the observation matrix's sequential properties and comprises 128 units with a tanh activation function. This layer takes the observation matrix as input and outputs the final hidden state, which is then fed into the second layer, a fully connected network with 12 units. Employing a scaled tanh activation function with a scaling factor of 0.3 ensures that output coefficients are mostly within $[-0.2, 0.2]$ μm, allowing the model to test its extrapolation capability beyond the training limit of 0.15 μm for each modal coefficient.

2. MOSS-DDPG training process and hyperparameters

The following algorithm describes the training process of MOSS-DDPG, inheriting the original DDPG structure [2] with refinement for the sensorless adaptive optics scenario.

Algorithm S1 MOSS-DDPG

Randomly initialize critic-network and actor-network with weights
Initialize Memory with a sufficient size to accommodate training data
Define warm-up duration $\mathcal{W}$
for episode = 1, N **do**
    Receive observation matrix $\mathcal{M}$
    Acquire action C of Zernike coefficients from actor-network with observation matrix $\mathcal{M}$
    for e = 1, $\mathcal{E}$ **do**
        Initialize a random Zernike coefficient noise profile $\mathcal{N}_e$ for action exploration
        Acquire reward r_e by applying action with noise to the wavefront
        Restore $[\mathcal{M}, C + \mathcal{N}_e, r_e]$ in Memory
    end for
    while episode > $\mathcal{W}$ **do**
        Sample a random batch of S transitions $(\mathcal{M}_i, C_i, r_i)$ from the Memory
        Divide the batch into mini-batches of size S_b
        for t = 1, S/S_b **do**
            Update critic network by minimizing the loss: $L = \frac{1}{S_b} \sum_i (r_i - Q(\mathcal{M}_i, C_i|\theta^Q))^2$
        end for
        for t = 1, S/S_b **do**
            Update the actor policy using the sampled policy gradient:
            $\nabla_{\theta^\mu} J \approx \frac{1}{S_b} \sum_i \nabla_C Q(\mathcal{M}, C|\theta^Q)|_{\mathcal{M}=\mathcal{M}_i, C=\mu(\mathcal{M}_i)} \nabla_{\theta^\mu} \mu(\mathcal{M}|\theta^\mu)|_{\mathcal{M}_i}$
        end for
    end while
end for

The corresponding hyperparameters for the *in-silico* MOSS-DDPG model exhibited in the manuscript are presented in Table. S3.

Table S3. MOSS-DDPG training hyperparameters in simulation

Hyperparameter Type	Value
Training Episodes N	40,000
Warm-up Episodes $\mathcal{W}$	4,000
Noise Profiles per Episode $\mathcal{E}$	15
Critic-network Learning Rate	0.0025
Actor-network Learning Rate	0.00025
Sampled Batch Size S	8,192
Mini-batch Size S_b	512
Maximum Gaussian Noise Standard Deviation	0.06 μm
Minimum Gaussian Noise Standard Deviation	0.001 μm
Number of Episodes Annealing	40,000
Maximum Memory Size	600,000

The hyperparameters for the *in-situ* MOSS-DDPG transfer learning process are presented in Table. S4.

Table S4. MOSS-DDPG training hyperparameters for *in-siu* transfer learning

Hyperparameter Type	Value
Training Episodes N	1,000
Warm-up Episodes $\mathcal{W}$	100
Noise Profiles per Episode $\mathcal{E}$	7
Critic-network Learning Rate	0.002
Actor-Network Learning Rate	0.0002
Sampled Batch Size S_b	128
Mini-batch Size S_b	32
Maximum Gaussian Noise Standard Deviation	0.03 μm
Minimum Gaussian Noise Standard Deviation	0.001 μm
Number of Episodes Annealing	1,000
Maximum Memory Size	7,000

Definitions of training episodes, warm-up steps, annealing Gaussian exploration noise, and sampled batch size inherit the original DDPG algorithm [2]. Several clarifications are provided

below for the specific scenario of MOSS-DDPG for sensorless adaptive optics.

Firstly, the "noise profiles per episode" is an exploration-enhancing technique for the single-step setting of MOSS-DDPG. This hyperparameter represents the number of noise profiles generated to be added to the action in one episode. All actions with different noise profiles are applied to the deformable mirror to acquire corresponding image metric values to be restored in the memory for training.

Secondly, the large sampled batch for each network update process is divided into mini-batches according to the "mini-batch size" hyperparameter. This division improves the frequency of network updates. Tuning this hyperparameter can enhance the stability and robustness of the training process, effectively balancing the exploration and exploitation speed of the DDPG agent.

For training MOSS-DDPG in correcting a different number of modal coefficients than 12, the hyperparameters should be adjusted accordingly. More training episodes and warm-up steps are generally expected for a larger number of modal coefficients.

References

1. S. Hochreiter and J. Schmidhuber, “Long short-term memory,” Neural Comput. **9**, 1735–1780 (1997).
2. T. P. Lillicrap, J. J. Hunt, A. Pritzel, *et al.*, “Continuous control with deep reinforcement learning,” (2019).
